# Prediction of lung dose‐volume parameters of the patients with esophageal cancer undergoing radiotherapy based on artificial neural network

**DOI:** 10.1002/acm2.70340

**Published:** 2025-11-10

**Authors:** Fahui Li

**Affiliations:** ^1^ Department of Radiotherapy the First Affiliated Hospital Fujian Medical University Fuzhou China; ^2^ Department of Radiotherapy National Regional Medical Center Binhai Campus of the First Affiliated Hospital Fujian Medical University Fuzhou China; ^3^ Key Laboratory of Radiation Biology of Fujian Higher Education Institutions The First Affiliated Hospital Fujian Medical University Fuzhou China

**Keywords:** artificial neural network, esophageal cancer, lung dose‐volume parameter prediction, radiotherapy

## Abstract

**Objective:**

Considering the complex and nonlinear relationship between characteristic parameters of tumors (location and morphology) and lung dose‐volume parameters of patients with esophageal cancer (EPC) undergoing intensity‐modulated radiation therapy (IMRT), the model identification method of artificial neural network (ANN) was adopted to build the model to predict lung dose‐volume parameters under different characteristic parameters of tumors. The goal is to enhance the efficiency and quality of radiotherapy planning and reduce the risk of radiation‐induced lung injury (RILI) by providing pre‐planning dose predictions.

**Methods:**

1). In the previous research work, a retrospective analysis was done on 103 cases of patients with EPC who were treated by radiotherapy, in which a linear regression model was employed and had proved the association between tumor morphology [including tumor relative length (*L*) and “tumor axial cross‐sectional area” (*S*)] and lung dose‐volume parameters (including *V*_5_, *V*_10_, *V*_20_ and *V*_30_ of lungs). 2). Building on earlier research, this study uses curve regression analysis to further explore the effect of tumor relative position (*P*) on lung dose‐volume parameters of patients with EPC undergoing IMRT. 3). A backpropagation (BP) neural network model was constructed with *P*, *L*, and *S* as inputs and lung dose‐volume parameters as outputs to establish their quantitative relationship. 4). Genetic algorithm (GA) was combined with BP neural network to optimize the initial weights and thresholds of BP neural network and avoid the model from falling into local optima, so as to improve the performance of BP neural network model.

**Results:**

1). Quadratic regression models were applied to analyze the relationship of *P* with *V*_5_ and *V*_10_ of the lung, yielding adjusted *R* ^2^ values of 0.177 and 0.081, respectively, with statistically significant regression coefficients (*p* < 0.01). The results suggest that as *P* increases, *V*_5_ and *V*_10_ initially increase, then decrease. Linear regression models were applied to analyze the relationship of *P* with *V*_20_ and *V*_30_ of the lung, yielding adjusted *R* ^2^ values of 0.06 and 0.072, respectively, with significant regression coefficients (*p* < 0.01). The results show that both *V*_20_ and *V*_30_ decrease as *P* increases. 2). The finding of this study indicates that the modeling method of BP neural network can realize the prediction of lung dose‐volume parameters under different characteristic parameters of tumors. The GA‐BP model showed slight improvements in prediction accuracy (*PA*) and error index (*EI*) compared to the BP model alone. For both models, prediction accuracy is highest for *V*_5_, followed by *V*_10_ and *V*_20_, with *V*_30_ being the least accurate. Notably, both the BP and GA‐BP neural networks achieve outstanding recognition accuracy for *V*_5_, with *EI* and *PA* values of 0.121 and 88.61% for BP, and 0.113 and 89.00% for GA‐BP, respectively. 3). The re‐optimization of four test cases with under‐predicted whole‐lung *V*_5_ and *V*_10_ values resulted in substantial improvements over the original plans. Conclusion: The ANN model developed in this study can effectively predict lung dose‐volume parameters for patients with EPC undergoing IMRT. It provides pre‐planning guidance that enhances the efficiency and quality of radiotherapy planning and reducing the risk of RILI.

## 1 INTRODUCTION

Cancer is a leading cause of premature death globally. Among various cancers, esophageal carcinoma (EPC) ranks as the eighth most common and is the sixth leading cause of cancer‐related mortality.^1^ Radiotherapy is crucial in the treatment of EPC, whether used after surgery or as the primary local therapy for patients with unresectable tumors. Its primary objective is to optimize therapeutic outcomes by achieving a higher probability of tumor control while minimizing complications in normal tissues.^2^ Intensity‐modulated radiation therapy (IMRT) offers improved target coverage and a greater potential to reduce complication rates compared to three‐dimensional conformal radiotherapy.^3^, ^4^ However, due to the close anatomical proximity of the esophagus to lungs, one of the most radiation‐sensitive organs, radiotherapy for patients with EPC inevitably leads to some degree of damage to the surrounding lungs, which can severely affect patient health and quality of life.

The reported incidence of clinically significant radiation‐induced lung injury (RILI) varies considerably across the existing literature. In patients receiving radiotherapy for malignant tumors located in the chest and mediastinum, the incidence rates have been reported to range from approximately 5% to 25%.^5^, ^6^ RILI is irreversible and adversely impacts treatment outcomes and prognosis, potentially leading to respiratory failure or even death in severe cases.^7^, ^8^ RILI has long been considered a major restrictive factor in the radiotherapy for patients with EPC.

The incidence of RILI is predominantly affected by clinical factors and dosimetric parameters of lungs,^9^, ^10^, ^11^ such as patient age, lung function, chemotherapy regimens, and different lung dose‐volume parameters. Currently, the likelihood of RILI is commonly assessed using lung dose‐volume parameters, which are widely acknowledged as critical indicators for pre‐evaluating the risk of RILI, supported by extensive research.^12^, ^13^ The lung dose‐volume parameters most commonly used are *V*_5_, *V*_10_, *V*_20_, and *V*_30_, which represent the volume percentages of the lung exposed to doses exceeding 5, 10, 20, and 30 Gy, respectively.

Radiotherapy planning is a core process during the treatment of patients with cancer. Before designing a radiotherapy plan, it is essential to determine the prescribed dose for the target area of tumors and the dose limits for organs at risk. Optimization conditions are determined based on clinical goals. Through continuous iterative optimization and adjustments, a radiotherapy plan that meets clinical requirements is ultimately achieved. This iterative process typically demands a substantial investment of time and manpower. Conversely, clinicians typically establish the prescribed dose for the target area of tumors and the dose limits for critical organs based on radiotherapy guidelines. Most of these guidelines are formulated using average values derived from historical data, which may not adequately address the individualized needs of patients in clinical practice. As a result, this can lead to guidelines that are either too strict or too lenient. Therefore, developing a personalized lung dose‐volume parameter prediction model is essential for guiding the formulation of radiotherapy plans. This approach will enable physicists to make informed clinical decisions in advance, enhancing the efficiency and quality of radiotherapy plans and reducing the incidence of RILI after treatment.

Previous research^14^ established “tumor axial cross‐sectional area” as a novel parameter for characterizing tumor morphology of patients with EPC. The geometric topology of tumor target volumes in patients with EPC was characterized using two parameters: tumor relative length and “tumor axial cross‐sectional area”. The linear regression analysis revealed a correlation between these morphological parameters of tumors (including tumor relative length and “tumor axial cross‐sectional area”) and lung dose‐volume parameters for patients with EPC during IMRT. Building on earlier research, in this study, we use curve regression analysis to further explore the effect of tumor relative position on lung dose‐volume parameters and have confirmed their association. Then, we comprehensively analyze the effects of tumor relative position, tumor relative length, and “tumor axial cross‐sectional area” on lung dose‐volume parameters, with the objective of developing a predictive model for lung dose‐volume parameters for patients with EPC undergoing IMRT. By providing physicists with predicted lung dose‐volume parameters before radiotherapy planning, this model seeks to enhance radiotherapy planning efficiency and quality, ultimately reducing the risk of RILI.

Given the complexity and nonlinearity of the relationship between tumor location and morphological parameters and lung dose‐volume parameters during IMRT for the patients with EPC, traditional mechanistic analysis methods often fail to establish precise theoretical mathematical models. Recent advancements in model identification techniques—such as artificial neural networks, fuzzy modeling, and support vector machines—have positioned system identification as a widely accepted and effective approach for modeling complex objects.^15^, ^16^, ^17^, ^18^ Artificial neural networks (ANNs) demonstrate excellent adaptability, self‐learning, self‐organization, and robust nonlinear fitting capabilities. This allows them to establish mapping relationships between input and output variables through supervised or unsupervised learning without the need for detailed mathematical models. Consequently, this study utilizes ANNs to develop a predictive model for lung dose‐volume parameters for the patients with EPC undergoing IMRT. This model is intended to provide theoretical guidance for the selection of optimization conditions for radiotherapy plans, assisting physicists in making timely clinical decisions during radiotherapy planning. Ultimately, this approach aims to enhance the efficiency and quality of radiotherapy plan design and reduce the incidence of RILI post‐treatment.

## 2 MATERIALS AND METHODS

### 2.1 Clinical data collection and outlier exclusion

The preliminary study conducted a retrospective analysis of 103 cases of EPC treated with IMRT and confirmed the correlation between tumor morphological parameters (tumor relative length and “tumor axial cross‐sectional area”) and lung dose‐volume parameters (*V*_5_, *V*_10_, *V*_20_, and *V*_30_). The radiation prescribed dose was the same for all 103 enrolled patients. Detailed patient information and the research methodology are provided in reference 14.

Here, tumor relative length (*L*) represents the PTV‐C length normalized to lung length, calculated as *L* = (length of PTV‐C)/(lung length). Tumor cross‐sectional area (*S*) denotes the average axial cross‐sectional area of the PTV‐C, derived from *S* = (volume of PTV‐C)/(length of PTV‐C). In which, the abbreviation “PTV‐C” was intended to denote the planning target volume, where the “C” suffix denotes its derivation from margin expansion of the clinical target volume (CTV). Specifically, only the values of tumor length and volume taken from the part between the upper and lower boundary of both lungs in the thoracic cavity were included and considered.

To ensure the continuity and comparability of the sample data, this study utilizes EPC case data from previous research.^14^ Building upon the findings of previous study, the present research further explores the impact of tumor relative position on lung dose‐volume parameters of the patients with EPC undergoing IMRT. To this end, tumor relative position data were integrated into the original dataset. In this study, tumor relative position is denoted as “*P*”, defined as the distance from the center of PTV‐C to the upper edge of the lung, divided by the lung length. Among the enrolled cases, the maximum tumor relative position was 0.83, the minimum was 0.11, the median was 0.38, and the first and third quartiles were 0.32 and 0.49, respectively.

To ensure data quality and detect potential outliers, anomaly detection was performed on the dataset. Multivariate outliers were identified using the Mahalanobis distance method, resulting in the removal of seven data sets. Consequently, 96 data sets were included in the final analysis, using a significance level of 0.05 and a Mahalanobis distance threshold of 7.81. The data for the enrolled cases are presented in the Table 1 below:

**TABLE 1:** Summary of the patients’ clinical characteristics.

	Tumor‐specific parameters			Lung dose‐volume parameters (%)			
Case	*P*	*L*	*S*	*V*_5_	*V*_10_	*V*_10_	*V*_10_
1	0.39	0.78	19.35	65.38	46.90	22.08	10.94
2	0.35	0.70	13.31	57.94	38.79	13.77	4.79
3	0.32	0.63	15.64	61.18	48.59	22.30	9.10
4	0.24	0.47	18.58	39.57	30.67	17.46	7.32
…	…	…	…	…	…	…	…
96	0.28	0.81	16.08	74.83	51.54	18.49	8.12

### 2.2 Associations between tumor relative position and lung dose‐volume parameters

To investigate the relationship between tumor position (*P*) and lung dose‐volume parameters (*V*_5_, *V*_10_, *V*_20_, and *V*_30_), curve regression analysis was conducted. This approach is particularly effective for modeling nonlinear relationships when linear regression models may not capture the complexity of the associations. Several regression models—including linear, quadratic, cubic, and logarithmic—were tested to account for potential relationship types. The significance of each regression coefficient was evaluated using *p*‐values, with *p* < 0.05 indicating a statistically significant impact of the independent variable on the dependent variable. The model with the best fit was selected based on adjusted *R* ^2^ and the scatter plot data distribution, ensuring the most accurate representation of the relationships among variables.

### 2.3 Artificial neural network model identification

Artificial neural network (ANN) is a mathematical model of biological nervous system, which simulates its structure and the processing information function. The basic architecture of ANN usually involves input layer, hidden layer, and output layer. Each layer consists of computational units (artificial neurons). Based on the selected learning algorithm, the learning ability of ANN is achieved by adjusting the weights and thresholds.

In the learning phase, the output of the network is compared with the target value (results of experiments) and the computed error of the network is back‐propagated to the hidden layers. Then, the weights will be adjusted by using these propagated errors. The neural network can be thought of as a non‐linear mapping from input space to output space, which “learns” or discovers the relationships between input and output variables by adjusting weights and thresholds. Hornik's theorem demonstrates that multilayer feedforward networks with a single sufficiently‐sized hidden layer universally approximate any continuous function to arbitrary precision, irrespective of complexity.^19^

#### 2.3.1 Network mapping relationship

Building on previous research, this study thoroughly analyzes the effects of tumor relative position (*P*), tumor relative length (*L*), and “tumor axial cross‐sectional area” (*S*) on lung dose‐volume parameters of patients with EPC undergoing radiotherapy. We employ ANN algorithms to develop a predictive model for lung dose‐volume parameters. In this context, *P*, *L*, and *S* represent tumor relative position, tumor relative length, and “tumor axial cross‐sectional area”, respectively. Tumor relative position refers to the spatial location of the PTV‐C in relation to the lung, defined as: *P* = (distance from the center of PTV‐C to the upper edge of the lung)/(lung length). Tumor relative length indicates the length of the PTV‐C compared to the lung, defined as: *L* = (length of PTV‐C)/(lung length). The axial cross‐sectional area represents the average axial cross‐sectional area of the PTV‐C, defined as: *S* = (volume of PTV‐C)/(length of PTV‐C).

By utilizing *P*, *L*, and *S* as inputs for the neural network, and lung dose‐volume parameters *V*x (where *x* = 5, 10, 20, and 30) as outputs, we construct a three‐layer artificial neural network model. The network's mapping relationship can be expressed as:

(1) $$V_{x}=f\left(P,L,S\right)\;\left(x=5,10,20,30\right)$$

In this study, the prescribed radiation dose was identical for all 103 cases; therefore, it was excluded as an input parameter in the neural network model.

#### 2.3.2 Neural network structure

**FIGURE 1:**
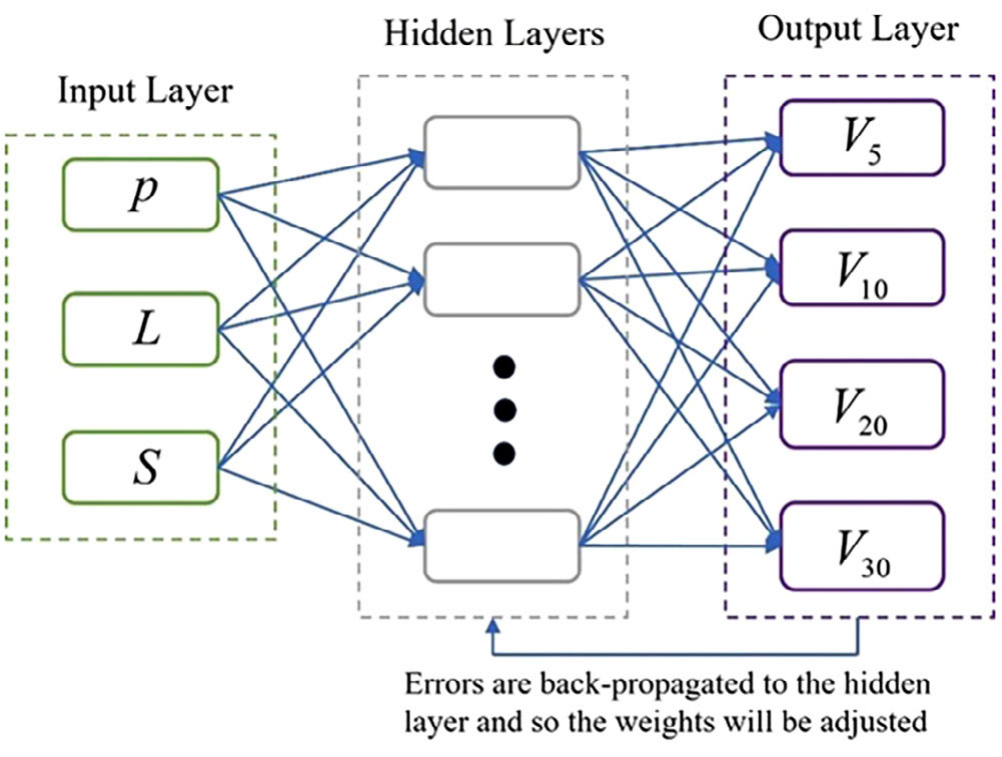
Schematic representation of the artificial neural network structure.

The ANN model was built based on the above‐mentioned mapping relationship between input and output variables. Its structure is shown in Figure 1.

As shown in Figure 1, the model involves an input layer, a hidden layer and an output layer with three input neurons in the input layer and four output neurons in the output layer. The input vector is *X* (X=[P,L,S]) and output vectors is *V*x.

Figure 1 depicts the structure of the developed ANN model, consisting of an input layer, a hidden layer, and an output layer. The input layer consists of three neural nodes, while the output layer comprises four neural nodes. These nodes correspond to the input variables *X *= [*P*, *L*, and *S*] and the output variables *V*x​ (where x = 5, 10, 20, and 30).

#### 2.3.3 Neural network training

96 cases were used for modeling and validation (excluding 7 cases). To evaluate the feasibility of the ANN model, we implemented a repeated fivefold cross‐validation strategy with five repetitions. Then, backpropagation (BP) neural network was adopted to establish the quantitative relationship between input and output variables.

During the network training process, the connection weight between the *j*‐th neuron and the *i*‐th neuron in the preceding layer is defined by Equation (2):

(2) $$w_{ij}\left(t+1\right)=w_{ij}\left(t\right)+\eta \delta_{j}{\tilde{y}}_{i}$$

In the above equation, *t* represents the number of training iterations; ${\tilde{y}}_{i}$​ denotes the output of the *i*‐th neuron; η is the learning rate; and $\delta_{j}$​ is the learning error of the neuron node, which is calculated using the following equation:

(3) $$\delta_{j}=\left\{\begin{array}{cc} {\tilde{y}}_{j}\left(1-{\tilde{y}}_{j}\right)\left(y_{j}-{\tilde{y}}_{j}\right)\; & j\;\text{is the output node}\\ {\tilde{y}}_{j}\left(1-{\tilde{y}}_{j}\right)\sum_{k=1}^{L}w_{jk}\left(t\right)\delta_{k}\;\;\; & j\;\text{is the hidden layer node} \end{array}\right.$$

Here, $y_{j}$ is the predicted output; ${\tilde{y}}_{j}$ is the actual output; $w_{jk}$ represents the connection weight between the *k*‐th neuron in the current layer and the *j*‐th neuron in the previous layer; and *L* denotes the number of nodes in the hidden layer.

#### 2.3.4 Improvement of artificial neural networks

**FIGURE 2:**
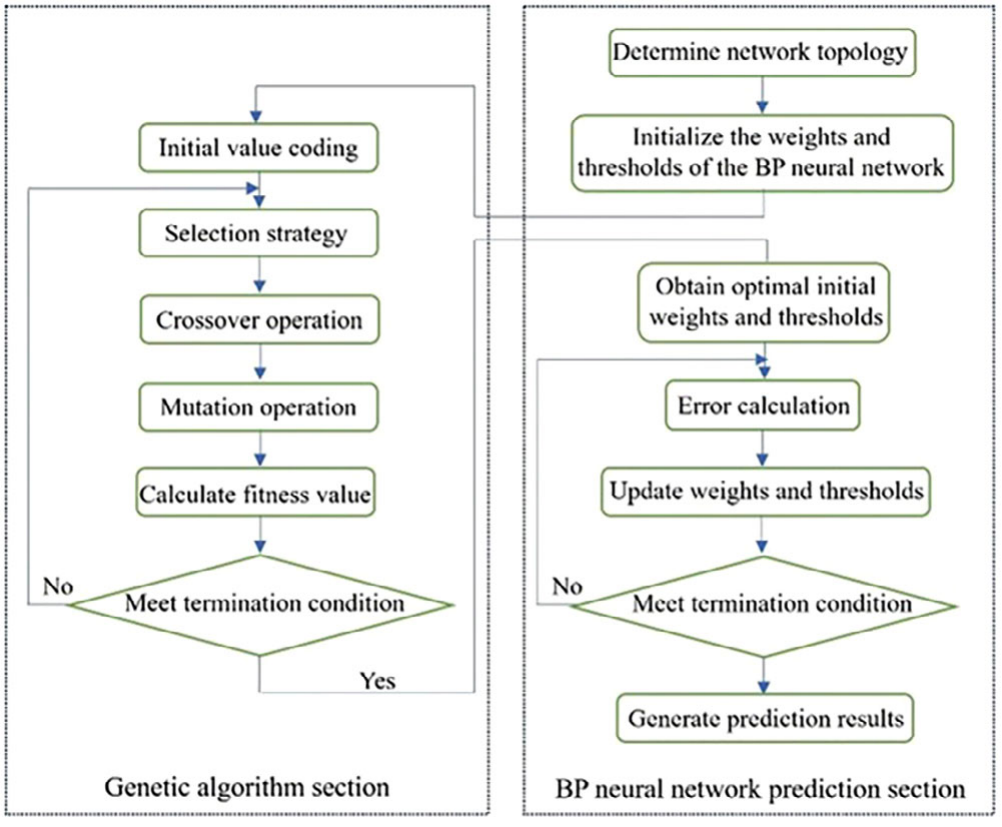
Flowchart for the optimization of backpropagation neural network model using genetic algorithm.

The BP neural network adjusts the weights and thresholds of neurons in each layer based on the errors at each neuron, progressively refining the predicted values to approximate the true values. This process establishes a mapping relationship between input and output variables. However, when training a feedforward multilayer neural network using the BP algorithm, the learning process is highly sensitive to the initial weights and thresholds due to the reliance on gradient descent. This sensitivity can cause the network to become trapped in local minima, resulting in slower convergence or even failure to converge. The genetic algorithm (GA) is an adaptive global optimization method inspired by the biological principles of “survival of the fittest” and “natural selection”. Unlike gradient descent, GA employs a global search strategy that does not depend on gradient information, making it less likely to become stuck in local optima and more likely to converge to the global optimum. Therefore, this paper integrates the genetic algorithm with the BP neural network to optimize its initial weights and thresholds, enhancing the performance of the traditional BP neural network. Figure 2 provides a flowchart illustrating the process of optimizing the BP neural network using the genetic algorithm.

## 3 RESULTS

### 3.1 Statistical findings on associations between tumor relative position and lung dose‐volume parameters

**FIGURE 3:**
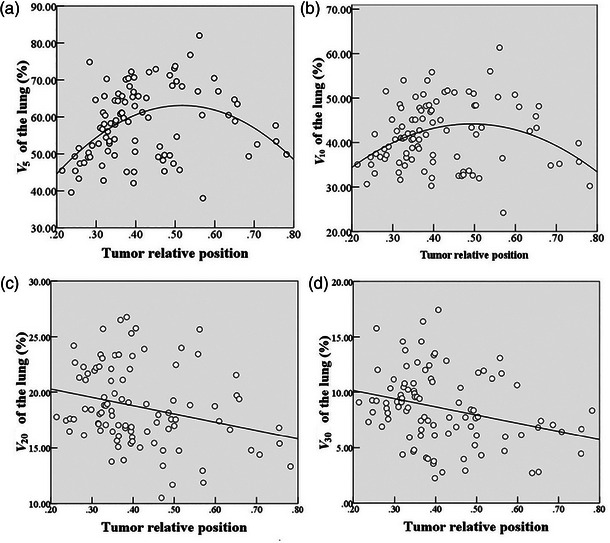
Scatter plot of tumor relative position and lung dose‐volume parameters (*V*_5_, *V*_10_, *V*_20_, and *V*_30_), with curve regression analysis applied.

This study utilizes various curve regression models to analyze the relationship between tumor relative position (*P*) and lung dose‐volume parameters (*V*_5_, *V*_10_, *V*_20_, and *V*_30_). Based on the adjusted *R* ^2^ and the scatter plot data distribution, the most suitable regression models were selected to ensure the best fit. For the dependent variables *V*_5_ and *V*_10_, quadratic regression models were applied, yielding adjusted *R* ^2^ values of 0.177 and 0.081, respectively. These values indicate that the models explain 17.7% and 8.1% of the variation in *V*_5_ and *V*_10_, with statistically significant regression coefficients (*p* < 0.01). The results suggest that as *P* increases, *V*_5_ and *V*_10_ initially rise and then decrease, as shown in Figure 3a,b.

For the dependent variables *V*_20_ and *V*_30_, linear regression models were employed, yielding adjusted *R* ^2^ values of 0.06 and 0.072, respectively. These values indicate that the models explain 6% and 7.2% of the variation in *V*_20_ and *V*_30_, with regression coefficients that are statistically significant (*p* < 0.01). The results reveal a decreasing trend in both *V*_20_ and *V*_30_ as *P* increases, as shown in Figure 3c,d.

### 3.2 Prediction of lung dose‐volume parameters

To evaluate the feasibility of the modified ANN model, this study employed a repeated fivefold cross‐validation strategy with five repetitions. A total of 96 cases were included and randomly partitioned into five mutually exclusive and approximately equal‐sized subsets. In each fold, four subsets were used for training and the remaining one for testing, resulting in five performance estimates per round. This entire fivefold cross‐validation procedure was repeated five times with different random splits to enhance the robustness of the evaluation. The final performance metric was calculated as the average of all 25 test results across the five repetitions.

The ANN model was constructed using a single hidden layer, with six nodes in the hidden layer. The training error of target parameter is taken as 1 × 10^−5^; the learning efficiency is set as η=0.1.

**FIGURE 4:**
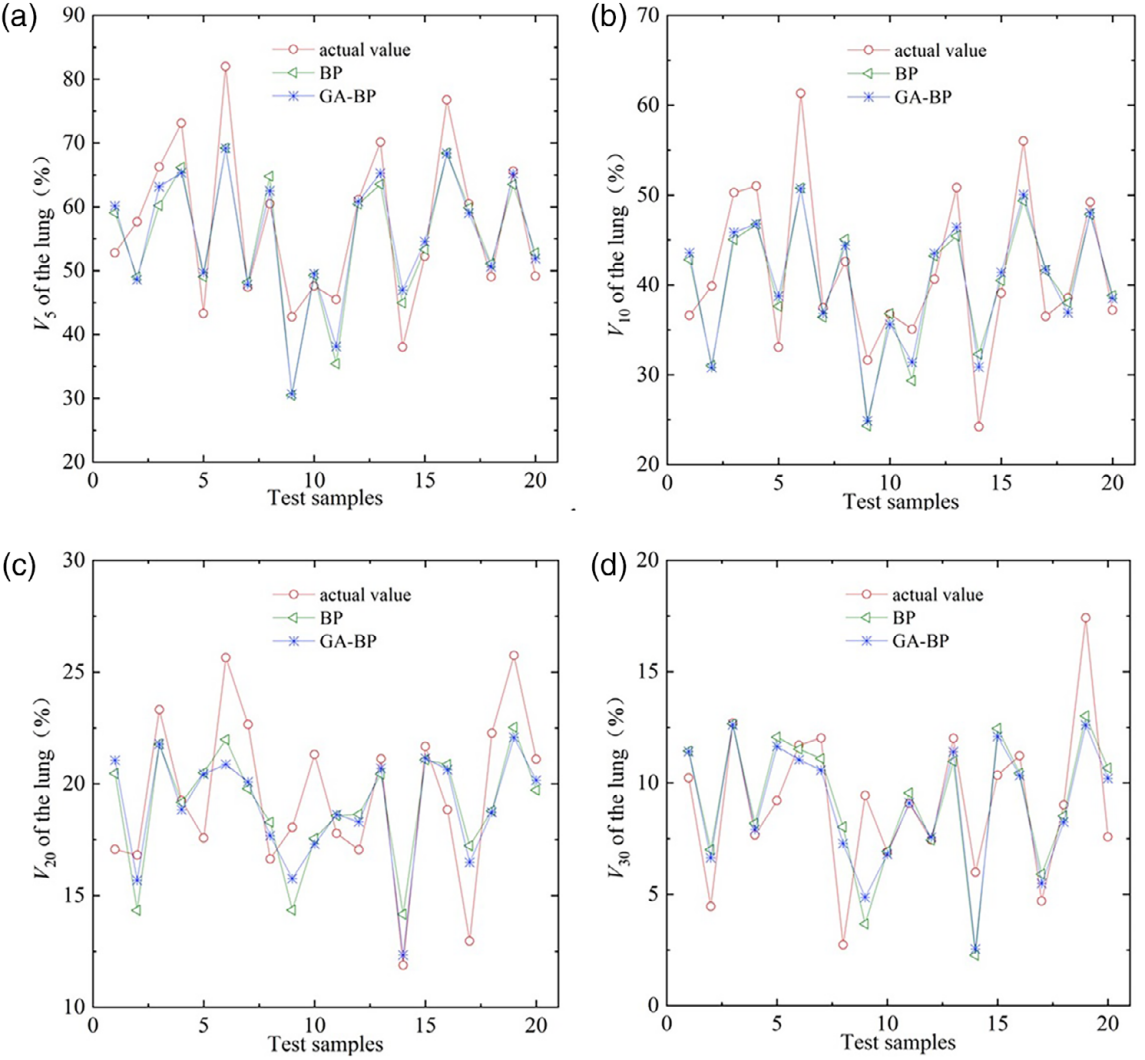
Comparison of identification results between the standard BP model and the BP model optimized by the genetic algorithm.

The identification results obtained by traditional BP neural network model and modified ANN (GA‐BP) model are shown in Figure 4. As demonstrated, the predictive model for lung dose‐volume parameters, developed using ANN, effectively captures the trends in lung dose‐volume parameters for patients with EPC undergoing IMRT, although some discrepancies still exist. In comparison, the GA‐BP neural network model produced results comparable to those from the standard BP neural network model, with no significant differences observed.

To evaluate the rationality of the model and the convergence of the learning algorithm, an index named the Error Index (*EI*) which indicated the fitting accuracy of the model was introduced. *EI* is defined as:

(4) $$EI=\sqrt{\frac{\sum {\left({\hat{S}}_{i}-S_{i}\right)}^{2}}{\sum {S_{i}}^{2}}}$$

Also, we defined a new indicator named the predictive accuracy (*PA*). It is considered to be accurate for the prediction when the relative error (abbreviated as η) of the test data sets is less than 0.2. *PA* can be expressed as:

(5) $$PA=\frac{n}{N}\times 100\%$$

Where, *N* is the total number of detected samples; *n* is the number of detected samples whose relative error is less than 0.2. In addition, η can be defined as:

(6) $$\eta_{i}=\left|\frac{{\hat{S}}_{i}-S_{i}}{S_{i}}\right|\;\;\left(i=1,2,\text{…}\right)$$

In equations (4) and (6), ${\hat{S}}_{i}$ and $S_{i}$ represent the identification value and experimental value for the ith test sample, respectively.

Under the same hyperparameter conditions, the *EI* and *PA* values obtained from 25 repeated runs were averaged. Table 2 presents the average *EI* and *PA* of the BP and GA‐BP models for predicting *V*_5_, *V*_10_, *V*_20_, and *V*_30_. All results were obtained using fivefold cross‐validation repeated five times. The results show that the GA‐BP neural network model achieves a slight improvement in *PA* and *EI* compared to the standard BP neural network. For both models, prediction accuracy is highest for *V*_5_, followed by *V*_10_ and *V*_20_, with *V*_30_ being the least accurate. Notably, both the BP and GA‐BP neural networks achieve outstanding recognition accuracy for *V*_5_, with *EI* and *PA* values of 0.121 and 88.61% for BP, and 0.113 and 89.00% for GA‐BP, respectively. For *V*_10_, the *EI* and *PA* values are 0.143 and 83.21% for BP, and 0.135 and 84.26% for GA‐BP.

**TABLE 2:** Performance comparison of BP and GA‐BP models.

		Predicted parameters			
Evaluation indicator	Model	*V*_5_	*V*_10_	*V*_20_	*V*_30_
*EI*	BP	0.121 ± 0.028	0.143 ± 0.029	0.162 ± 0.020	0.276 ± 0.038
	GA‐BP	0.113 ± 0.023	0.135 ± 0.024	0.164 ± 0.017	0.256 ± 0.036
*PA*/%	BP	88.61 ± 7.09	83.21 ± 7.55	75.12 ± 9.50	53.73 ± 12.28
	GA‐BP	89.00 ± 6.73	84.26 ± 7.10	73.64 ± 8.34	53.67 ± 9.44

### 3.3 Efficacy of predictive lung dose‐volume parameters in guiding radiotherapy plan optimization

**FIGURE 5:**
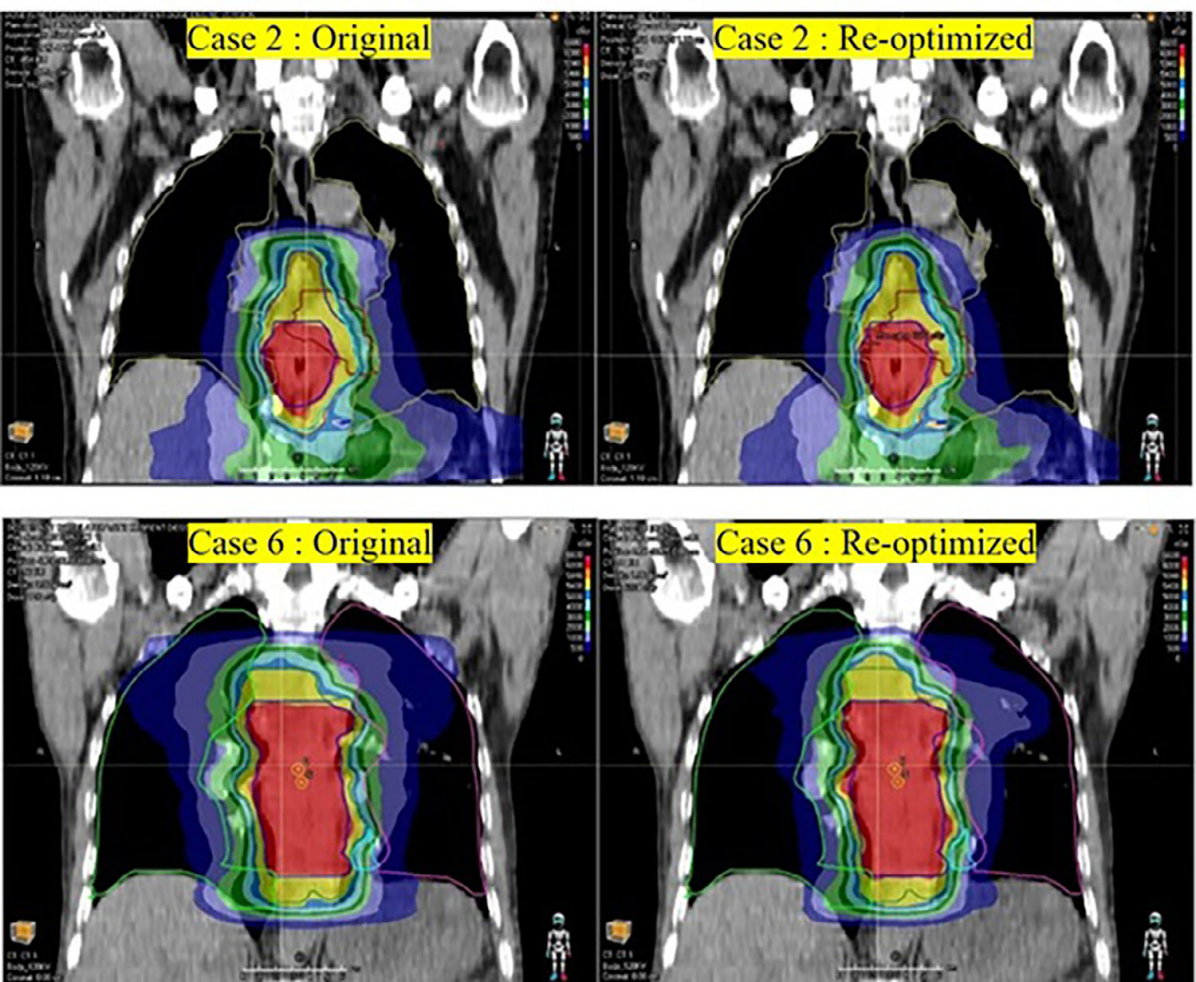
Comparison between the original and re‐optimized radiotherapy plans for Case 2 and Case 6.

As shown in Figure 4a,b, the model‐predicted values of *V*_5_ and *V*_10_ of lungs for cases 2, 6, 9, and 11 were significantly lower than the actual values, indicating potential optimization opportunities for these parameters. Accordingly, the radiotherapy plans for the four cases were re‐optimized, and their dose distributions were compared against the original plans. The comparison results are shown in Table 3 and Figure 5, 6. The results demonstrated that the re‐optimized plan significantly reduced the *V*_5_ and *V*_10_ of lungs while maintaining target coverage and avoiding dose increases to other organs at risk, such as the spinal cord and heart. In Table 3, the conformity index (*CI*) is computed using the formula:

(7) $$CI=\left(V_{D\_PTV}/V_{D}\right)\times \left(V_{D\_PTV}/V_{PTV}\right)$$

where *V*_PTV_ is the volume of the planning target volume (PTV), *V*_D_PTV_ represents the volume of the PTV receiving the prescribed dose, and *V*_D_ is the total volume receiving the prescribed dose. A *CI* value closer to 1 indicates a more conformal treatment plan.

**TABLE 3:** Comparison of original radiotherapy plans, model predictions, and re‐optimized plans for cases 2, 6, 9, and 11.

		Lungs		Heart	SC‐PRV	
Case	Status	*V*_5_/%	*V*_10_/%	*D*_mean_/Gy	*D*_max_/Gy	*CI*
2	Original	57.65	39.87	32.31	44.01	0.84
	Predicted	48.58	30.81	∖	∖	∖
	Re‐optimized	49.29	31.52	31.35	36.52	0.80
6	Original	82.00	61.34	33.98	44.42	0.82
	Predicted	69.19	50.72	∖	∖	∖
	Re‐optimized	69.79	48.69	32.45	43.65	0.80
9	Original	42.79	31.62	4.44	48.36	0.77
	Predicted	30.68	24.88	∖	∖	∖
	Re‐optimized	34.83	25.27	3.73	44.09	0.76
11	Original	45.49	35.07	2.01	44.97	0.79
	Predicted	38.11	31.38	∖	∖	∖
	Re‐optimized	39.38	28.96	1.49	44.01	0.83

Abbreviations: *CI*, conformity index; *D*_max_, maximum dose; *D*_mean_, mean dose; SC‐PRV, spinal cord planning risk volume.

**FIGURE 6:**
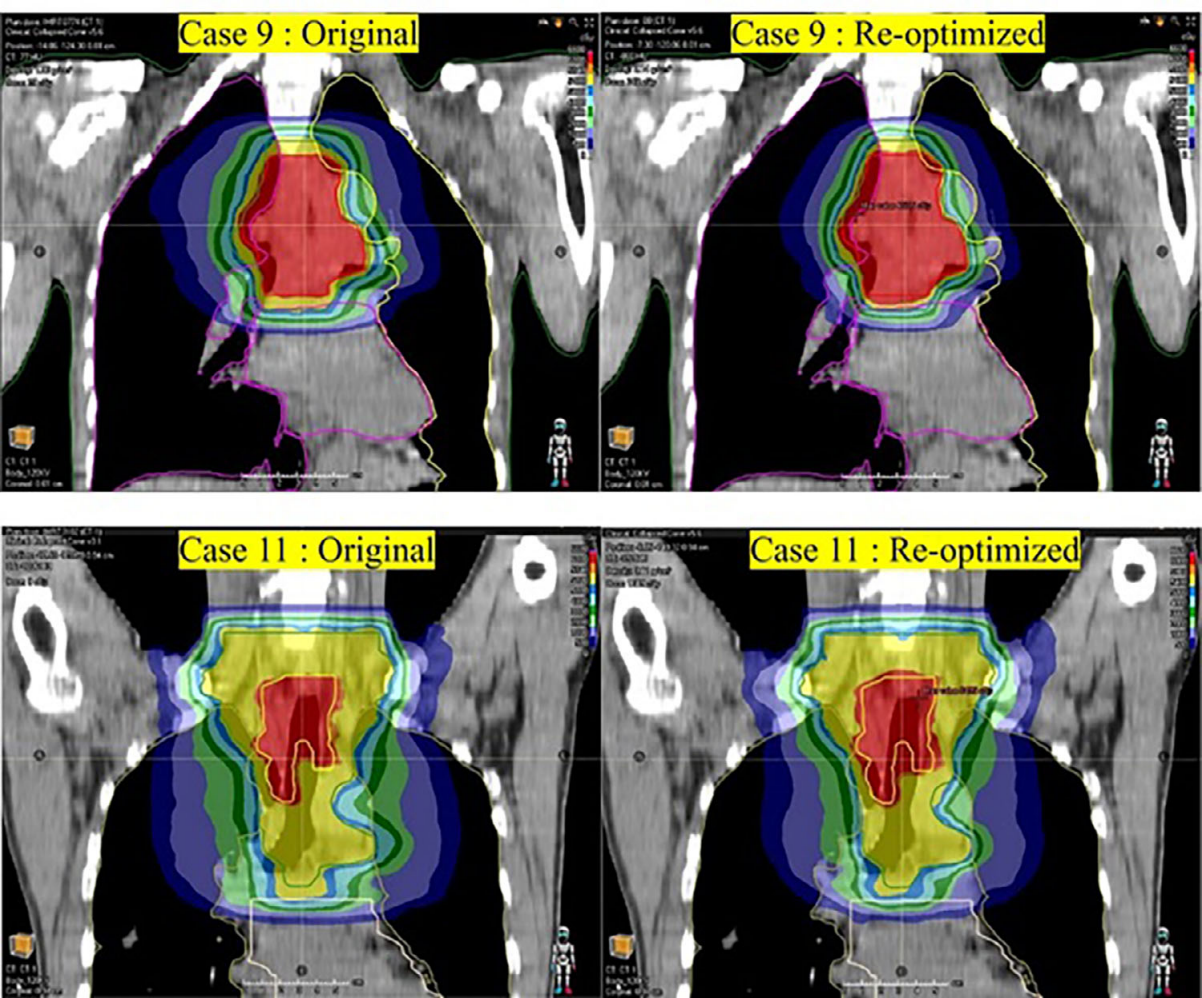
Comparison between the original and re‐optimized radiotherapy plans for Case 9 and Case 11.

## 4 DISCUSSION

In previous research^14^ the association between tumor morphology [including tumor relative length (*L*) and “tumor axial cross‐sectional area” (*S*)] and lung dose‐volume parameters (including *V*_5_, *V*_10_, *V*_20_ and *V*_30_ of lungs) was established. In this study, we further demonstrate the significant associations between tumor relative position (*P*) and lung dose‐volume parameters in patients with EPC undergoing IMRT. Quadratic regression models were applied to analyze the relationship of *P* with *V*_5_ and *V*_10_ of the lung, yielding adjusted *R* ^2^ values of 0.177 and 0.081, respectively, with statistically significant regression coefficients (*p* < 0.01). The results suggest that as *P* increases, *V*_5_ and *V*_10_ initially increase, then decrease. Linear regression models were applied to analyze the relationship of *P* with *V*_20_ and *V*_30_ of the lung, yielding adjusted *R* ^2^ values of 0.06 and 0.072, respectively, with significant regression coefficients (*p* < 0.01). The results show that both *V*_20_ and *V*_30_ decrease as *P* increases.

It can be seen from the results that tumor relative position (*P*) has a greater impact on the low‐dose region of the lung. As *P* increases, *V*_5_ and *V*_10_ first increase and then decrease, whereas *V*_20_ and *V*_30_ consistently decrease. The reasons may be as follows: (i) As the tumor shifts toward the middle part of the lung, more lung tissue is affected by the radiation dose, resulting in an increase in the value of the lung dose‐volume parameters, especially for *V*_5_ and *V*_10_ of the lung. (ii) The high‐dose region in the lung is primarily influenced by the volume of lung tissue adjacent to the tumor. As the tumor shifts toward the lower part of the lung, the presence of the heart between the lower lung segments reduces the amount of lung tissue near the tumor. This reduction in adjacent lung volume contributes to a decrease in the high‐dose region, thereby leading to a reduction in *V*_20_ and *V*_30_.

Based on the above research foundation, we comprehensively analyze the effects of tumor relative position, tumor relative length, and “tumor axial cross‐sectional area” on lung dose‐volume parameters, with the objective of developing a predictive model for lung dose‐volume parameters, so as to improve the efficiency and quality of radiotherapy planning and reduce the occurrence of RILI by providing the predicted lung dose‐volume parameter for the physicist before radiotherapy planning. The results show that the GA‐BP neural network model achieves a slight improvement in *PA* and *EI* compared to the standard BP neural network. For both models, prediction accuracy is highest for *V*_5_, followed by *V*_10_ and *V*_20_, with *V*_30_ being the least accurate. Notably, both the BP and GA‐BP neural networks achieve outstanding recognition accuracy for *V*_5_, with *EI* and *PA* values of 0.121 and 88.61% for BP, and 0.113 and 89.00% for GA‐BP, respectively. For *V*_10_, the *EI* and *PA* values are 0.143 and 83.21% for BP, and 0.135 and 84.26% for GA‐BP.

From a clinical perspective, the findings of this study are highly significant. By providing physicists with predictions of lung dose‐volume parameters ahead of radiotherapy planning, the need for repetitive adjustments is reduced, ultimately enhancing both the efficiency and quality of the planning process. During the radiotherapy planning process, when the calculated value of lung dose‐volume parameters exceeds the predicted value obtained from the model developed in this study by a certain margin, this indicates potential optimization opportunities for these parameters. Radiotherapy physicists can further reduce the values of lung dose‐volume parameters to decrease the incidence of RILI.

Nevertheless, the model's limitations should be acknowledged. While demonstrating promising accuracy for *V*_5_ (89.00%) and *V*_10_ (84.26%), its predictive performance for *V*_20_ (75.12%) and *V*_30_ (53.73%) remains clinically suboptimal for precision‐sensitive applications. Given that all radiotherapy plans were generated within the RayStation treatment planning system, measurement and delivery errors were unlikely to significantly contribute to the observed deviations of the models. We attribute these deviations primarily to three factors: (i) The model did not incorporate detailed anatomical information of tumors or organs‐at‐risk beyond the following specified inputs: tumor relative position, tumor relative length, and “tumor axial cross‐sectional area”. (ii) Plan optimization relied heavily on the physicists’ clinical experience, introducing inter‐ and intra‐planner inconsistencies. (iii) The size, diversity and quality of the dataset are pivotal in determining the performance of predictive models.

In conclusion, this study demonstrates the potential of ANN in predicting lung dose‐volume parameters of patients with EPC undergoing IMRT, offering new perspectives for radiotherapy planning in patients with EPC. Future research should focus on expanding datasets, refining modeling algorithms, and integrating detailed anatomical information to enable more precise and personalized predictive models. With these efforts, machine learning technologies can play an increasingly central role in radiation oncology, providing patients with improved treatment options and outcomes.

## 5 CONCLUSION

Building upon earlier research, this study employed nonlinear regression analysis to further investigate the influence of tumor relative position (*P*) on lung dose‐volume parameters in patients with EPC undergoing IMRT, confirming a statistically significant association between these variables. The results revealed a nonlinear relationship for *V*_5_ and *V*_10_, which initially increase and then decrease as *P* increases, while *V*_20_ and *V*_30_ demonstrated a consistent decline with increasing *P*.

An artificial neural network (ANN) model was developed to predict lung dose‐volume parameters in EPC patients receiving IMRT. The model addresses complex nonlinear relationships between tumor characteristics and radiation exposure by incorporating tumor relative position, tumor relative length, and “tumor axial cross‐sectional area” as inputs, with lung dose‐volume parameters as outputs. Results demonstrate that the GA‐BP neural network model achieves a slight improvement in *PA* and *EI* compared to the standard BP neural network. For both models, prediction accuracy is highest for *V*_5_, followed by *V*_10_ and *V*_20_, with *V*_30_ being the least accurate. Notably, both the BP and GA‐BP neural network models achieve outstanding recognition accuracy for *V*_5_, with *EI* and *PA* values of 0.121 and 88.61% for BP, and 0.113 and 89.00% for GA‐BP, respectively. For *V*_10_, the *EI* and *PA* values are 0.143 and 83.21% for BP, and 0.135 and 84.26% for GA‐BP.

In conclusion, these results demonstrate the ANN model's clinical viability for predicting lung dose‐volume parameters before radiotherapy planning for patients with EPC undergoing IMRT. By providing radiotherapy physicists with pre‐planning predictions of lung dose‐volume parameters, our approach reduces the need for repetitive adjustments, thereby enhancing both the efficiency and quality of the planning process. During treatment planning, if the calculated lung dose‐volume parameters exceed model predictions by a clinically significant margin, this suggests potential opportunities for further optimization. In such cases, physicists can proactively adjust the plan to reduce these parameters and lower the risk of RILI.

## AUTHOR CONTRIBUTIONS

**Fahui Li**: Conception and design; methodology; collection and assembly of data; data analysis and interpretation; software; model establishment and validation; manuscript writing and editing; final approval of manuscript.

## CONFLICT OF INTEREST STATEMENT

The authors declare no conflicts of interest.

## ETHIC STATEMENT

This retrospective study was approved by the ethics committee of the First Affiliated Hospital of Fujian Medical University [(2024)544].

## Data Availability

The data that support the findings of this study are available from the corresponding author upon reasonable request.
